# Engineering and Applications of fungal laccases for organic synthesis

**DOI:** 10.1186/1475-2859-7-32

**Published:** 2008-11-20

**Authors:** Adinarayana Kunamneni, Susana Camarero, Carlos García-Burgos, Francisco J Plou, Antonio Ballesteros, Miguel Alcalde

**Affiliations:** 1 Departamento de Biocatálisis, Instituto de Catálisis y Petroleoquímica, CSIC, Marie Curie 2, 28049 Madrid, Spain

## Abstract

Laccases are multi-copper containing oxidases (EC 1.10.3.2), widely distributed in fungi, higher plants and bacteria. Laccase catalyses the oxidation of phenols, polyphenols and anilines by one-electron abstraction, with the concomitant reduction of oxygen to water in a four-electron transfer process. In the presence of small redox mediators, laccase offers a broader repertory of oxidations including non-phenolic substrates. Hence, fungal laccases are considered as ideal green catalysts of great biotechnological impact due to their few requirements (they only require air, and they produce water as the only by-product) and their broad substrate specificity, including direct bioelectrocatalysis.

Thus, laccases and/or laccase-mediator systems find potential applications in bioremediation, paper pulp bleaching, finishing of textiles, bio-fuel cells and more. Significantly, laccases can be used in organic synthesis, as they can perform exquisite transformations ranging from the oxidation of functional groups to the heteromolecular coupling for production of new antibiotics derivatives, or the catalysis of key steps in the synthesis of complex natural products. In this review, the application of fungal laccases and their engineering by rational design and directed evolution for organic synthesis purposes are discussed.

## Laccases: general features

### Distribution

Laccases (benzenediol:oxygen oxidoreductase, EC 1.10.3.2) belong to the multicopper oxidase family, along with such different proteins as plant ascorbic oxidase, mammalian ceruloplasmin or Fet3p ferroxidase from *Saccharomyces cerevisiae*, among others [1]. These copper-containing enzymes catalyze the oxidation of various substrates with the simultaneous reduction of molecular oxygen to water [2]. Yoshida first discovered laccases in 1883 after observing that latex from the Japanese lacquer tree (*Rhus vernicifera*) hardened in the presence of air [3,4]. This makes laccase as one of the oldest enzymes ever described. Since then, laccase activity has been found in plants, some insects [5,6], and few bacteria [7]. However, most biotechnologically useful laccases (i.e. those with high redox potentials) are of fungi origin. Over 60 fungal strains belonging to Ascomycetes, Deuteromycetes and especially Basidiomycetes show laccase activities. Among the latter group, white-rot fungi are the highest producers of laccases but also litter-decomposing and ectomycorrhizal fungi secret laccases [8].

### Biochemical features

Laccases are typically monomeric extracellular enzymes containing four copper atoms bound to 3 redox sites (T1, T2 and T3). The termed "blue copper" at the T1 site-because of its greenish-blue colour in its oxidized resting state-is responsible of the oxidation of the reducing substrate. The trinuclear cluster (containing one Cu T2 and two Cu T3) is located approx. 12 Å away from the T1 site, and it is the place where molecular oxygen is reduced to water [1]. Laccases catalyze one-electron substrate oxidation coupled to the four-electron reduction of O_2_. It is assumed that laccases operate as a battery, storing electrons from the four individual oxidation reactions of four molecules of substrate, in order to reduce molecular oxygen to two molecules of water.

Fungal laccases often occur as multiple isoenzymes expressed under different cultivation conditions (e.g. inducible or constitutive isoforms). Most are monomeric proteins, although laccases formed by several units have been also described [9,10]. They are glycoproteins with average molecular mass of 60–70 kDa, and carbohydrate contents of 10–20% which may contribute to the high stability of laccases. The covalently linked carbohydrate moiety of the enzyme is typically formed by mannose, N-acetylglucosamine and galactose. The amino acid chain contains about 520–550 amino acids including a N-terminal secretion peptide [4].

### Biological functions and industrial applications

Biological functions attributed to laccases include spore resistance and pigmentation [11,12], lignification of plant cell walls [13], lignin biodegradation, humus turnover and detoxification processes [8], virulence factors [12], and copper and iron homeostasis [14].

**Figure 1:**
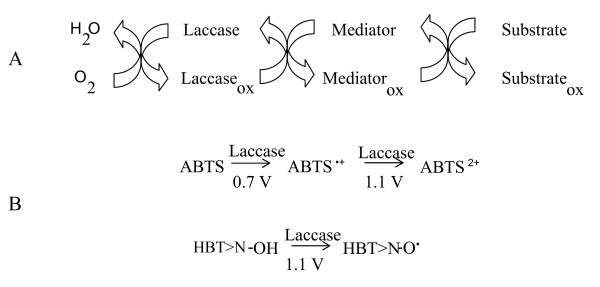
Expanded role of laccase oxidizing non-usual substrates by the action of redox mediators (A); and redox potentials of the oxidation reactions of ABTS and HBT by laccase (B).

Laccases exhibit an extraordinary natural substrate range (phenols, polyphenols, anilines, aryl diamines, methoxy-substituted phenols, hydroxyindols, benzenethiols, inorganic/organic metal compounds and many others) which is the major reason for their attractiveness for dozens of biotechnological applications [15-17]. Moreover, in the presence of small molecules, known as redox mediators, laccases enhance their substrate specificity. Indeed, laccase oxidizes the mediator and the generated radical oxidizes the substrate by mechanisms different from the enzymatic one, enabling the oxidative transformation of substrates with high redox potentials-otherwise not oxidized by the enzyme-, Figure 1A. The industrial applicability of laccase may therefore be extended by the use of a laccase-mediator system (LMS). Thus, laccase and LMS find potential application in delignification and biobleaching of pulp [18-21]; treatment of wastewater from industrial plants [22,23]; enzymatic modification of fibers and dye-bleaching in the textile and dye industries [24,25]; enzymatic crosslinking of lignin-based materials to produce medium density fiberboards [26]; detoxification of pollutants and bioremediation [27-31]; detoxification of lignocellulose hydrolysates for ethanol production by yeast [32,33]; enzymatic removal of phenolic compounds in beverages-wine and beer stabilization, fruit juice processing [34-36]-; and construction of biosensors and biofuel cells [37].

In organic synthesis, laccases have been employed for the oxidation of functional groups [38-42], the coupling of phenols and steroids [43-45], the construction of carbon-nitrogen bonds [46] and in the synthesis of complex natural products [47] and more.

As mentioned above, many of these applications require the use of redox mediators opening a big window for new biotransformations of non-natural substrates towards which laccase alone hardly shows activity. On the other hand, in most of the cases large quantities of enzymes are required, which makes the efficient expression of laccase in heterologous systems an important issue. Moreover, the protein engineering of fungal laccases with the aim of improving several enzymatic features (such as activity towards new substrates, stability under harsh operating conditions -*e.g. *presence of organic cosolvents, extreme pH values-, thermostability, and others) is a critical point in the successful application of this remarkable biocatalyst. All these issues are addressed in the following lines, paying special attention to their application in organic synthesis.

## Laccase-mediator system (LMS)

The combination of the laccase with low molecular weight molecules such as 2,2'-azino-bis-(3-ethylbenzothiazoline-6-sulphonic acid) (ABTS) or 1-hydroxybenzotriazole (HBT) not only lead to higher rates and yields in the transformation of laccase substrates but also add new oxidative reactions to the laccase repertory towards substrates in which the enzyme alone had no or only marginal activity, Figure 1A, B. Thus, LMS enlarges substrate range being able to oxidize compounds with redox potential (E°) higher than that of laccase (typically, laccase E° at the T1 site is in the range +475 to +790 mV but the LMS allows to oxidize molecules with E° above +1100 mV) [48,49]. Besides, the mediator acts as a diffusible electron carrier enabling the oxidation of high molecular weight biopolymers such as lignin, cellulose or starch [1]. Hence, the steric issues that hinder the direct interaction between enzyme and polymer are overcome by the action of the redox mediator.

**Figure 2:**
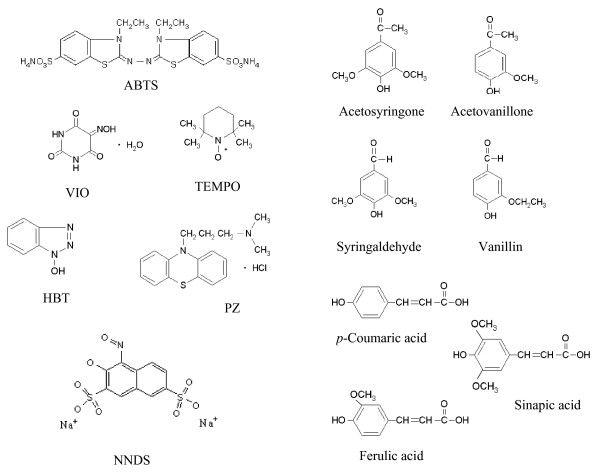
Chemical structures of some representative artificial (ABTS, HBT, violuric acid -VIO-, TEMPO, promazine -PZ- and 1-nitroso-naphthol-3,6-disulfonic acid -NNDS-) and lignin-derived natural mediators (acetosyringone, syringaldehyde, vanillin, acetovanillone, *p*-coumaric acid, ferulic acid and sinapic acid).

LMS has resulted highly efficient in many biotechnological and environmental applications as regards the numerous research articles and invention patents published [50,51]. Many artificial mediators have been widely studied, from ABTS the first described laccase mediator [52], to the use of synthetic mediators of the type -NOH- (such as HBT, violuric acid (VIO), N-hydroxyphtalimide (HPI) and N-hydroxyacetanilide (NHA), the stable 2,2,6,6-tetramethyl-1-piperidinyloxy free radical (TEMPO), or the use of phenothiazines and other heterocycles (*e.g. *promazine or 1-nitroso-naphthol-3,6-disulfonic acid), Figure 2[18,38,53]. More recently, complexes of transition elements (polyoxometalates) have been also demonstrated to mediate lignin degradation catalyzed by laccase [54,55].

**Figure 3:**
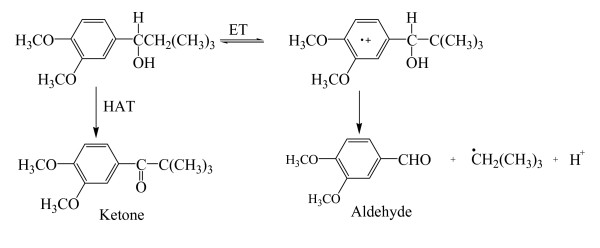
**Diagram showing the differences between the oxidation mechanisms followed by ABTS radicals (Electron Transfer route, ET) and HBT radicals (Hydrogen Atom Transfer route, HAT) in LMS for oxidation of non-phenolic substrates (according to Galli and Gentili**[52]**).**

**Figure 4:**
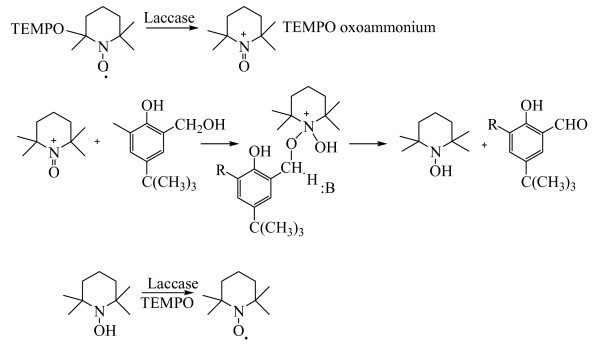
**Mechanisms of the laccase-TEMPO oxidation of hydroxymethyl groups to aldehyde groups by TEMPO according to d'Acunzo et al. **[43].

The choice of a proper mediator (over 100 redox mediators have been described [56]) represents a key consideration for a given biotransformation. The use of different mediators may yield different final products when using the same precursors. This is basically due to the fact that substrate oxidation in laccase-mediator reactions occurs via different mechanisms. The mediator radicals preferentially perform a specific oxidation reaction based on its chemical structure and effective redox potential (or dissociation bond energy) [43,38,53,57]. For example, ABTS and HBT follow two different radical pathways: i) electron transfer (ET) in the case of ABTS radicals (ABTS^•+^or ABTS^2^^+^) and ii) hydrogen atom transfer (HAT) for nitroxyl radicals (N-O^•^) of HBT, Figure 3. On the contrary, the stable radical TEMPO follows an ionic oxidation mechanism [38,39], Figure 4.

Despite all the associated advantages of LMS, there are two major drawbacks hindering the use of mediators: they are expensive and they can generate toxic derivatives. Moreover, in some cases, while oxidizing the mediator, laccase is inactivated by the mediator radicals, or the latter can be transformed into inactive compounds with no more mediating capability (*e.g. *generation of benzotriazol from HBT by losing the hydroxyl group). Last trends are focusing in the use of low-cost and eco-friendly alternative mediators; in this sense, several naturally occurring mediators produced by fungi (phenol, aniline, 4-hydroxybenzoic acid and 4-hydroxybenzyl alcohol) have been identified [49]. More recently, phenolic compounds derived from lignin degradation (such as acetosyringone, syringaldehyde, vanillin, acetovanillone, ferulic acid or *p*-coumaric acid) have been demonstrated to be highly-efficient laccase mediators of natural origin (even better than the powerful artificial ones) for dye decolorization, removal of polycyclic aromatic hydrocarbons, pulp bleaching and pitch removal [58-61], Figure 2. These natural compounds can be obtained at low cost due to their abundance in nature and also in industrial paper pulp wastes, smoothing the progress to a more environmental-friendly and sustainable white biotechnology processes.

## Heterologous expression of fungal laccases

Biotechnological and environmental applications require large amounts of enzymes. Laccases secreted from wild-type fungal organisms may not be suitable for commercial purposes mainly because the low yields and undesirable preparation procedures (such as presence of toxic inducers) are not economically advantageous; however recent advances in bioreactor design and culture conditions have significantly increased the production yields [62].

Heterologous expression should be better suited for large-scale production, because of the potential of expressing different laccases in one selected optimised host. Laccases, like other oxidative enzymes, are difficult to express in non-fungal systems. The heterologous expression of active laccases has been reported mainly in filamentous fungi (*Aspergillus oryzae*, *Aspergillus niger, Aspergillus sojae *and *Trichoderma reseei*) and yeasts (*Saccharomyces cerevisiae, Pichia pastoris*, *Pichia methalonica*, *Yarrowia lipolytica *and *Kluyveromyces lactis*), Table 1. There is one remarkable exception of homologous expression, in which the basidiomycete fungus *Pycnoporus cinabarinus *was used as host to overexpress the active laccase (up to 1.2 g l^-1^) [63]. Unfortunately, the functional expression of fungal laccases in bacteria (*Escherichia coli*) has not been yet accomplished (perhaps due to the requirement of glycosylation, missing chaperones, and different codon usage, among other shortcomings).

**Table 1:** List of heterologously expressed laccases

**Laccase**	**Source**	**Host**	**Comments**	**References**
PO1	*Coriolus hirsutus*	*Saccharomyces cerevisiae*	Active laccase secreted in the medium.	Kojima et al. [64]
PO2			Active laccase secreted in the medium.	Kojima et al. [64]

PrL	*Phlebia radiata*	*Trichoderma reesei*	Laccase secreted activity of 7.7 nkat ml^-1 ^(ABTS). The enzyme was purified and partially characterized.	Saloheimo and Niku-Paavola [65]

LCC1, LCC4	*Rhizoctonia solani*	*Aspergillus oryzae*	Laccase activity secreted in the medium. The enzyme was purified and partially characterized.	Wahleithner et al. [66]
LCC2			Active laccase secreted in the medium.	Wahleithner et al. [66]

LCC1	*Trametes villosa*	*Aspergillus oryzae*	Active laccase secreted in the medium. The enzyme was purified and partially characterized.	Yaver et al. [9]

MtL	*Myceliophtora thermophila*	*Aspergillus oryzae*	Laccase secreted activity of 0.85 U ml^-1 ^(SGZ). The enzyme was purified and partially characterized.	Berka et al. [67]
		*Saccharomyces cerevisiae*	Laccase secreted activity of 0.6 U l^-1 ^(ABTS). Total activity was enhanced 170-fold by directed evolution (18 mg l^-1^).	Bulter et al. [68]

LCC1	*Trametes versicolor*	*Pichia pastoris*	Active laccase secreted in the medium. Production yield was further optimised.	Jönsson et al. [69]; O'Callaghan et al. [70]; Hong et al. [71]
LCC1		*Saccharomyces cerevisiae*	Undetectable laccase activity in the medium.	Cassland and Jönsson [72]
LCC2		*Saccharomyces cerevisiae*	Active laccase secreted in the medium. Production of ethanol from raw materials (0.12 U l^-1^).	Cassland and Jönsson [72] Larsson et al. [73]
LCCI		*Pichia pastoris*	Active laccase secreted in the medium. The enzyme and a truncated version (LCCIa) were purified and partially characterized.	Gelo-Pujic et al. [74]
LCCIV		*Pichia pastoris*	Laccase secreted activity of 0.15 U ml^-1 ^(ABTS). The enzyme was purified and partially characterized.	Brown et al. [75]
LCCI		*Zea mays L*	Laccase activity was found in the seed, and variability in the amount was seen. The highest level was 0.55% TSP (respect to total soluble protein).	Hood et al. [76]
LCC1		*Pichia methalonica*	9.79 U ml^-1 ^of laccase acivity in recombinant with the α-factor signal peptide.	Guo et al. [77]
LACIIIb		*Yarrowia lipolytica*	2.5 mg l^-1 ^(0.23 U ml^-1^) of active enzyme with limited excess of glycosylation.	Jolivalt et al. [78]
LCCα		*Saccharomyces cerevisiae*	0.035 U l^-1 ^of laccase activity produced by *S. cerevisiae.*	Necochea et al. [79]
LCC1, LCC2		*Pichia pastoris Aspergillus niger*	2.8 U l^-1 ^of laccase activity produced by *P. pastoris *and up to 2700 U l^-1 ^by *A. niger.*	Bohlin et al. [80]
Gene IV		*Aspergillus niger*	592 U l^-1 ^of enzyme activity in solid-state fermentation produced by *A. niger*.	Téllez-Jurado et al. [81]

LAC	*Schizophyllum commune*	*Aspergillus sojae*	Laccase secreted activity of 774 U ml^-1 ^(Gallic acid).	Hatamoto et al. [82]

LCC1	*Coprinus cinereus*	*Aspergillus oryzae*	Transformants secreted from 8.0 to 135 mg of active laccase per liter. The enzyme was purified and partially characterized.	Yaver et al. [83]

LCC1	*Coprinopsis cinerea*	*Coprinopsis cinerea*	Maximal activity (3 U ml^-1^) reached with the *gpdII *promoter and 0. 1 μM CuSO_4 _(homologous expression).	Kilaru et al. [84]

LtLACC2	*Liriodendron tulipifera*	*Tobacco cells*	Protoplasts retained laccase activity which could be measured once the protoplasts were lysed.	LaFayette et al. [85]
LAC1	*Pycnoporus cinnabarinus*	*Pichia pastoris*	Transformants secreted 8.0 mg l^-1 ^of hyperglycosylated active laccase.	Otterbein et al. [86]
LAC1		*Aspergillus niger*	70 mgl^-1 ^of active laccase using the A. *niger *signal peptide which represent a 77-fold increased activity (7000 U ml^-1^) (ABTS). The enzyme was purified and partially characterized.	Record et al. [87]
LAC 1		*Aspergillus oryzae*	80 mgl^-1 ^of active laccase.	Sigoillot et al. [88]
LAC 1		*Pycnoporus cinnabarinus*	Laccase secreted activity of 1200 mg l^-1 ^(homologous expression)	Alves et al. [63]
LAC 1		*Yarrowia lipolytica*	20 mg l^-1 ^of active enzyme in bioreactor.	Madzak et al. [89]

LAC2	*Loblolly pine (Pinus taeda)*	*Saccharomyces cerevisiae*	Yeast cells accumulated the expected fusion protein in insoluble fractions without degradation of products, but no laccase activity was detected.	Sato et al. [90]

PPOA	*Marinomonas mediterranea*	*Escherichia coli*	Production of recombinant protein, with the most of activity, located in the membrane fraction rather than in the soluble one.	Sanchez-Amat et al. [91]

LAC4	*Pleurous sajor-caju*	*Pichia pastoris*	Transformants produced 4.85 mg l^-1 ^of active laccase. The enzyme was purified and partially characterized.	Soden et al. [92]

PPO	*Solanum tuberosum L.*	*Lycopersicon esculentum*	Active laccases secreted in the medium conferring resistance to pathogen *Pseudomonas syringae *pv *tomato*.	Li and Steffens [93]

LAC 1	*Melanocarpus albomyces*	*Trichoderma reesei*	920 mg L l^-1 ^of active laccase	Kiiskinen et al. [94]
LAC 1		*Saccharomyces cerevisiae*	168 U l^-1 ^of laccase activity produced (around 3 mg l^-1^)	Kiiskinen et al. [94]

LAC3	*Trametes sp. strain C30*	*Saccharomyces cerevisiae*	2 mg l^-1 ^of rLAC3 produced in bioreactor.	Klonowska et al. [95]

POXA1b, POXC	*Pleurotus ostreatus*	*Kluyveromyces lactis Saccharomyces cerevisiae*	*K. lactis *was more effective host (1.1 of POXA1b and 1.4 mg l^-1 ^of POXC laccase) than *S. cerevisiae*.	Piscitelli et al. [96]
3M7C mutant		*Saccharomyces cerevisiae*	~30 mU OD600 l^-1 ^after 6 days of incubation in shaken flask.	Festa et al. [97]
POXA3		*Kluyveromyces lactis*	80 U l^-1 ^after 10 days of incubation.	Faraco et al. [98]

LCC1	*Pycnoporus coccineus*	*Aspergillus oryzae Saccahromyces cerevisiae*	High copper concentrations are required for the production of active laccase.	Hoshida et al. [99]

LCC1	*Coprinopsis cinerea*	*Coprinopsis cinerea*	Maximal activity (3 U ml^-1^) reached with the *gpdII *promoter and 0. 1 μM CuSO_4_	Kilaru et al. [84]

LCC	*Tametes trogii*	*Pichia pastoris*	17 mg l^-1 ^of active enzyme, reaching up to 2520 U l^-1 ^in fed-batch culture.	Colao et al. [100]
LCC1		*Kluyveromyces lactis*	6.6 U l^-1 ^of bioactive molecule produced by *K. lactis.*	Camattari et al. [101]

LACB	*Trametes sp.*	*Pichia pastoris*	Overexpression (1.01 U/mg) of active laccase (32000 U ml^-1^).	Li et al. [102]

LACD	*Trametes sp 420*	Pichia pastoris	8.3 × 10^4 ^U l^-1+^of active laccase.	Hong et al. [103]

Ery3	*Pleurotus eryngii*	*Aspergillus niger*	Partially characterization of recombinant laccase.	Rodríguez et al. [104]
Pel3		*Saccharomyces cerevisiae*	139 mU ml^-1 ^of laccase in alginate immobilized cells and 18°C.	Bleve et al. [105]

LCC	*Fome lignosus*	*Pichia pastoris*	3.7-fold expression improvement (up to 144 mg l^-1^) with EMS random mutagenesis.	Hu et al. [106]

## Laccase engineering

Crystallographic structure determination is an essential tool for structure-function relationships studies (*i.e. *rational design). However, since the crystallization of the first (but inactive) laccase from *Coprinus cinereus *in 1998 by Ducros *et al*.[107], few crystal structures of active laccases have been published: one from the ascomycete *Melanocarpus albomyces *[108], two from basidomycetes *Trametes versicolor *[109] and *Rigidosporus lignosun *[110] and another from *Bacillus subtilis *[111]. Based on these laccase structures, over the last decade several residues in the neighbourhood of the catalytic copper ions have been subjected to site-directed mutagenesis to determine the parameters that define the catalytic activity and the E° of fungal laccases [112,113]. One consequence of these comprehensive structure-function studies has been the generation of a collection of mutants with structural perturbations at the T1 copper center.

To overcome many of the limitations of the rational design, and in the absence of enough structural information, directed molecular evolution represents a promising alternative. This methodology recreates in the laboratory the key events of natural evolution (mutation, recombination and selection) doing in such a manner those more efficient enzymes-even with novel functions-can be tailored. Diversity is mimicked by inducing mutations and/or recombination in the gene encoding a specific protein. Afterwards, the best performers in each generation are selected and further used as the parental types for a new round of evolution. The process is repeated as many times as necessary enhancing exponentially the targeted features, until a biocatalyst with the desired traits is obtained: stability at high temperature or in organic solvents; improved catalytic activities; higher specificity; etc.

A thorough understanding of efficient and reliable high-throughput screening methodologies is a prerequisite for the design and validation of this type of experiments [114]. A key query result of *smart *laboratory evolution is the improvement of several enzymatic properties at the same time (e.g. stability and activity). The first successful example of directed laccase evolution reported came from Arnold group [68]. They carried out the functional expression of a thermophilic laccase in *S. cerevisiae *by directed evolution: after ten rounds of laboratory evolution and screening, the total enzymatic activity was improved 170-fold along with better performances at high temperatures.

It is well known that most of the laccase catalysed transformations for organic syntheses (from the oxidation of steroid hormones to the enzymatic polymerisation required for the synthesis of phenolic-based resins such as poly-α-naphtol, poly-pyrogallol and poly-catechol [1,115]., as well as conductive water-soluble polymers [116]) must be carried out in the presence of organic solvents. However, at high concentrations of organic co-solvents laccases undergo unfolding, therefore losing their catalytic activity. Recently, our group generated a thermostable laccase-the genetic product of five rounds of directed evolution expressed in *S. cerevisiae *[117,118]-that tolerates high concentrations of co-solvents. This evolved laccase mutant is capable of resisting a wide array of biotechnologically relevant miscible co-solvents at concentrations as high as 50% (v/v). Indeed, in 40% (v/v) ethanol or in 30% (v/v) acetonitrile the performance of the laccase mutant was comparable to that of the parental enzyme in aqueous solution, a capacity that has not been acquired in nature. Intrinsic electrochemical laccase features such as the redox potential at the T1 and T2/T3 sites and the geometry and electronic structure of the catalytic coppers varied slightly during the course of the *in vitro *evolution. Indeed, some mutations at the protein surface stabilized the evolved laccase by allowing additional electrostatic and hydrogen-bonding to occur [117]. Additionally, the protein folding in the post-translational maturation steps seemed to be modified by mutations in processing regions [119].

Besides methods that involve iterative steps of random mutagenesis and/or DNA recombination, semi-rational studies-which take advantage from both protein structure and combinatorial libraries constructed by saturation mutagenesis- are being employed successfully. This approach involves the mutation of any single amino acid codon to all the other codons that will generate the 20 naturally occurring amino acids coupled to screen for the desire function. This technique is commonly employed to improve the characteristics of enzymes at "*hot-spot*" residues already identified by conventional random mutagenesis. In addition, it can be employed to simultaneously mutate several codons (combinatorial saturation mutagenesis), which will enable all possible combinations of interesting residues to be evaluated in order to identify their optimal interactions and synergies.

In a recent study [120] of the evolved *Myceliophthora thermophila *laccase variant T2 (MtLT2) expressed in *S. cerevisiae *[68], we applied combinatorial saturation mutagenesis to residues L513 (the axial non-coordinating ligand supposedly essential for the E° at the T1 site) and S510 (belonging to the tripeptide _509_VSG_511 _that is common to the low-medium E° laccases). A mutant with 3-fold higher turnover rates than the parent type, contained one beneficial mutation (_TCG_S510G_GGG_) that could not be achieved by conventional error-prone PCR techniques, since it was dependent on the two consecutive nucleotide changes. In a more exhaustive study [119], several regions of the same variant were investigated by combinatorial saturation mutagenesis. After exploring over 180,000 clones, the S510G mutant revealed a direct interaction between the conserved _509_VSG_511 _tripeptide located in the neighbourhood of the T1 site and the C-terminal plug.

## Applications of laccases in organic synthesis

Organic synthesis of chemicals suffers from several drawbacks, including the high cost of chemicals, cumbersome multi-step reactions and toxicity of reagents [2,17]. Laccases might prove to be very useful in synthetic chemistry, where they have been proposed to be applicable for production of complex polymers and medical agents [16,121]. Indeed, the application of laccase in organic synthesis has arisen due to its broad substrate range, and the conversion of substrates to unstable free (cation) radicals that may undergo further non-enzymatic reactions such as polymerization or hydration. The list of laccases used for organic synthesis is presented in Table 2.

**Table 2:** List of laccases used for organic synthesis

**Laccase source**	**Application**	**Reference**
*Coriolus hirsutus*	Synthesis of an indamine dye	Baker et al. [122]
	Synthesis of conducting polyaniline	Karamyshev et al. [116]

*Pycnoporus cinnabarinus*	Synthesis of 3-(3,4-dihydroxyphenyl)-propionic acid derivatives	Mikolasch et al. [45]

*Pycnoporus coccineus*	Polymerization to functional polymers	Uyama and Kobayashi [123]

*Pyricularia oryzae*	Oxidative coupling of 3-methyl 2-benzothiazolinone hydrazone and methoxyphenols	Setti et al. [124]

*Trametes versicolor*	Synthesis of aromatic aldehydes	Fritz-Langhals and Kunath [40]
	Polymerization of 1-napthol	Akta et al. [125]
	Synthesis of substituted imidazoles and dimerization products	Schäfer et al. [126]
	Polymerization of catechol	Akta and Tanyolaç [127]
	Cross-linking of a protein	Boumans et al. [128]
	Synthesis of 3,4-dihydro-7,8-dihydroxy-2*H*-dibenzofuran-1-ones	Hajdok et al. [129]

*Trametes villosa*	Polymerization of bisphenol A	Uchida et al. [130]

*Trametes hirsuta*	Oligomerization of protein	Mattinen et al. [131]

*Trametes pubescens*	Oxidation of sugars derivatives	Marzorati et al. [132]
	Oxidation of natural glycosides	Baratto et al. [133]
	Synthesis of totarol	Ncanana et al. [134]

*Pyricularia oryzae*	Crosslinking of recombinant proteins	Suderman et al. [135]

*Agaricus bisporus*	Synthesis of 3,4-dihydro-7,8-dihydroxy-2*H*-dibenzofuran-1-ones	Hajdok et al. [129]

*Myceliophthora*	Synthesis of poly(catechin)	Kurisawa et al. [136]

### Laccases for enzymatic polymerization and polymer functionalization

Enzymatic polymerization using laccases has drawn considerable attention recently since laccase or LMS are capable of generating straightforwardly polymers that are impossible to produce through conventional chemical synthesis [127].

For example, the polymerization ability of laccase has been applied to catechol monomers for the production of polycatechol [127]. Polycatechol is considered a valuable redox polymer; among its applications are included chromatographic resins and the formation of thin films for biosensors. Former methods for the production of polycatechol used soybean peroxidase or horseradish peroxidase (HRP), which suffer from the common "suicide H_2_O_2 _inactivation". The main limitation of all heme-containing peroxidases is their low operational stability, mostly due to their rapid deactivation by H_2_O_2_-with half-lifes in the order of minutes in the presence of 1 mM H_2_O_2 _[127,137].

Inert phenolic polymers, for example poly(1-napthol), may also be produced by laccase-catalyzed reactions [125,138-140]. These polymers have application in wood composites, fiber bonding, laminates, foundry resins, abrasives, friction and molding materials, coatings and adhesives [125,141].

The enzymatic preparation of polymeric polyphenols by the action of laccases has been investigated extensively in the past decades as a viable and non-toxic alternative to the usual formaldehyde-based chemical production of these compounds [142-144]. Poly(2,6-dimethyl-1,4-oxyphenylene)-"poly(phenylene oxide)", PPO-, is widely used as high-performance engineering plastic, since the polymer has excellent chemical and physico-mechanical properties. PPO was first prepared from 2,6-dimethylphenol monomer using a copper/amine catalyst system. 2,6-Dimethylphenol was also polymerized through HRP catalysis to give a polymer consisting of exclusively 1,4-oxyphenylene units [145]. On the other hand, a small amount of Mannich-base and 3,5,3'5'-tetramethyl-4,4'-diphenoquinone units are contained in the commercially available PPO. The polymerization also proceeded under air in the presence of laccase derived from *Pycnoporus coccineus *without the addition of H_2_O_2 _[123,146].

**Figure 5:**
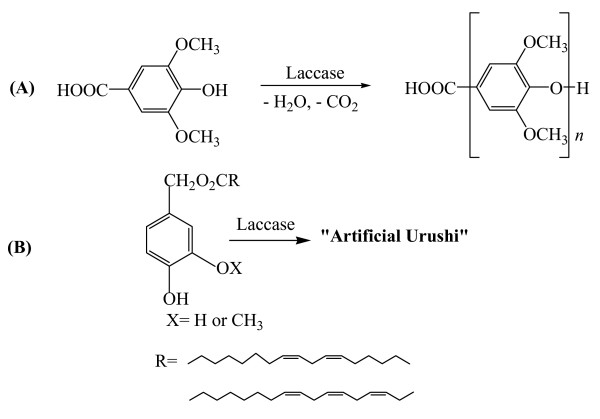
(A) PPO derivatives obtained from 4-hydroxybenzoic acid derivatives by laccase catalysis, and (B) "Artificial Urushi" prepared from new "urushiol analogues" by a laccase-catalyzed cross-linking reaction.

It has been also reported that laccase induced a new type of oxidative polymerization of 4-hydroxybenzoic acid derivatives, 3,5-dimethoxy-4-hydroxybenzoic acid (syringic acid) and 3,5-dimethyl-4-hydroxybenzoic acid. The polymerization involved elimination of CO_2 _and H_2 _from the monomer to give PPO derivatives with molecular weight up to 1.8 × 10^4 ^(Figure 5A) [145,147].

A novel system of enzymatic polymerization, i.e. a laccase-catalyzed cross-linking reaction of new "urushiol analogues" for the preparation of "artificial urushi" polymeric films (Japanese traditional coating) has been demonstrated (Figure 5B) [148-151]. Flavonoids have been also polymerized by polyphenol oxidase and laccase. The flavonoid-containing polymers showed good antioxidant properties and enzyme inhibitory effect [152].

It has been reported that laccase induced radical polymerization of acrylamide with or without mediator [146]. Laccase has been also used for the chemo-enzymatic synthesis of lignin graft-copolymers [153]. Along these lines, the potential of this enzyme for crosslinking and functionalizing lignocellulose compounds is also reported [154]. Laccases can be used in the enzymatic adhesion of fibers in the manufacturing of lignocellulose-based composite materials, such as fiber boards. In particular, laccase has been proposed to activate the fiberbound lignin during manufacturing of the composites, and boards with good mechanical properties without toxic synthetic adhesives have been obtained by using laccases [155,156]. Another possibility is to functionalize lignocellulosic fibers by laccases in order to improve the chemical or physical properties of the fiber products. Preliminary results have shown that laccases are able to graft various phenolic acid derivatives onto kraft pulp fibers [157,158]. This ability could be used in the future to attach chemically versatile compounds to the fiber surfaces, possibly resulting in fiber materials with completely novel properties, such as hydrophobicity or charge.

**Figure 6:**
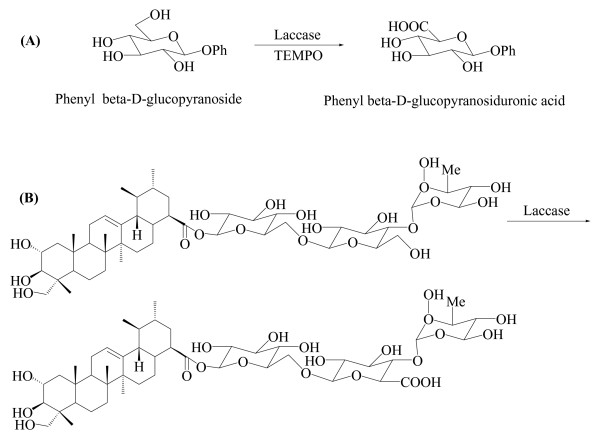
(A) Products obtained by the oxidation of sugars using laccase and TEMPO, and (B) enzymatic modification of the natural glycoside asiaticoside.

Finally, laccase-TEMPO mediated system has been also used to catalyze the regioselective oxidation of the primary hydroxyl groups of sugar derivatives or even starch, pullulan and cellulose allowing the polymer functionalization [132,159]. The efficiency of this system was initially tested with mono- and disaccharides (*i.e.*, phenyl β-D-glucopyranoside), and the corresponding glucopyranosiduronates were isolated and characterized (Figure 6A). Subsequently, this chemo-enzymatic approach has been exploited to achieve the partial oxidation of a water soluble cellulose sample. Also, the same approach has been applied for the mild oxidation of the glycosylated saponin, asiaticoside [160] (Figure 6B), and a series of natural glycosides [133].

### Oxidative transformation of organic compounds by laccase

**Figure 7:**
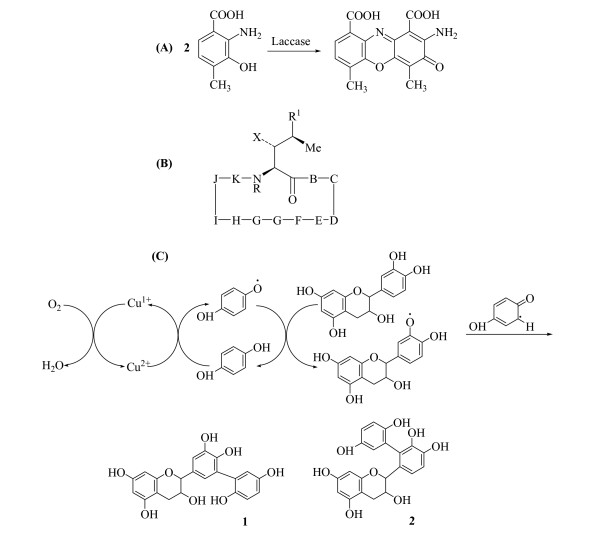
(A) Synthesis of actinocin via a laccase-catalyzed reaction, (B) Synthesis of novel cyclosporin reaction product obtained from cyclosporin A by HBT-mediated laccase oxidation, (C) Products obtained by the laccase/hydroquinone-mediated oxidation of (+)-catechin.

Laccases have been used to synthesize products of pharmaceutical importance. The first chemical that comes to mind is actinocin, synthesized via a laccase-catalyzed reaction from 4-methyl-3-hydroxyanthranilic acid as shown in Figure 7A. This pharmaceutical product has proven effective in the fight against cancer as it blocks transcription of tumor cell DNA [161,162].

Other examples of the potential application of laccases for organic syntheses include the oxidative coupling of katarantine and vindoline to yield vinblastine. Vinblastine is an important anti-cancer drug, especially useful in the treatment of leukemia. Vinblastine is a natural product that may be extracted from the plant *Catharanthus roseus*. The compound is however only produced in small quantity in the plant, whereas the precursors-namely katarantine and vindoline- are at much higher concentrations, and thus are relatively inexpensive to obtain and purify. A method of synthesis has been developed through the use of laccase with preliminary results reaching 40% conversion of the precursors to vinblastine [2]. Laccase coupling has also resulted in the production of several other novel compounds that exhibit beneficial properties, e.g. antibiotic properties [163].

The study of new synthetic routes to aminoquinones is of great interest because a number of antineoplast drugs in use, like mitomycin, or under development, like nakijiquinone-derivatives [164] or herbamycin-derivatives [165], contain an aminoquinone moiety. Several simple aminoquinones possess activity against a number of cancer cell-lines [166-168] as well as antiallergic or 5-lipoxygenase inhibiting activity [168,169].

Laccases have also been employed to synthesize new cyclosporin derivatives [170]. Cyclosporin A was converted to cyclosporin A Methyl vinyl ketone [R^1 ^= (E)-2-butenyl to R^1 ^= (E)-3-oxo-1-butenyl] by HBT-mediated laccase oxidation [170], (Figure 7B).

Laccases are also able to oxidize catechins. These molecules are the condensed structural units of tannins, which are considered important antioxidants found in herbs, vegetables and teas. Catechins ability to scavenge free radicals makes them important in preventing cancer, inflammatory and cardiovascular diseases. Oxidation of catechin by laccase has yielded products (Figure 7C) with enhanced antioxidant capability [136,171].

**Figure 8:**
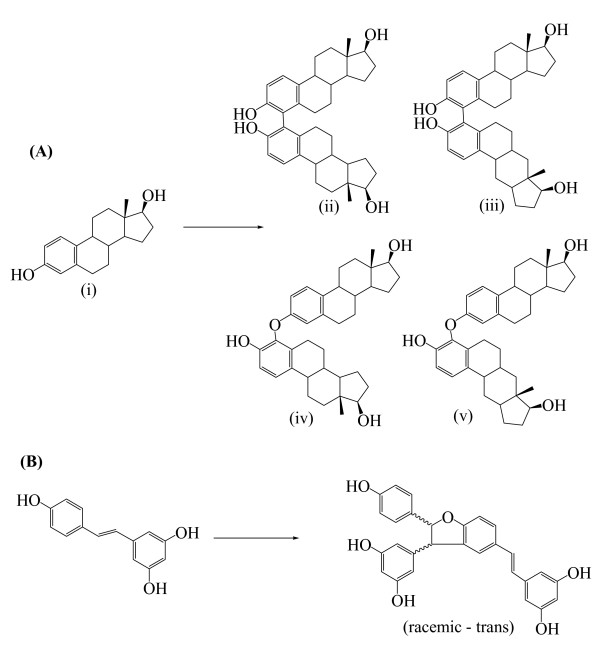
(A, ii-v) Dimeric products obtained by the oxidation of β-estradiol, (B) Dimeric product obtained by the oxidation of the phytoalexin resveratrol.

**Figure 9:**
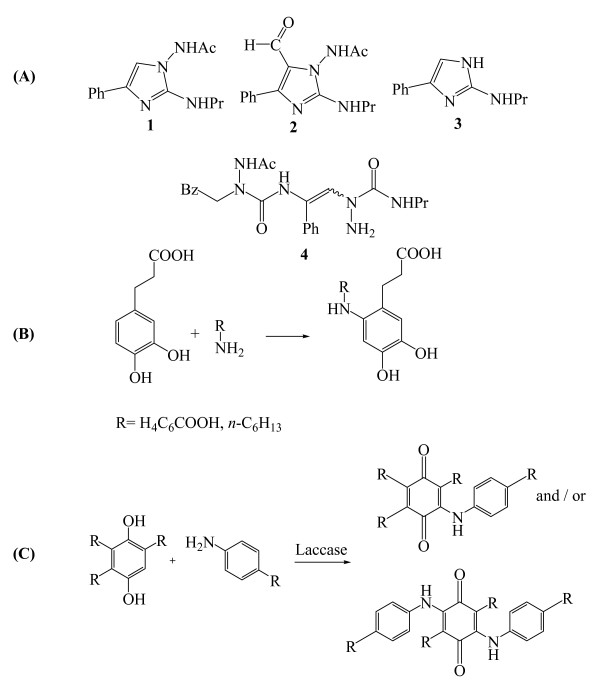
(A) *N*-[2-alkylamino-4-phenylimidazol-1-yl]-acetamide (substrate 1) and products 2–4 formed during incubation with *T. versicolor *laccase, (B) The natural compound 3-(3,4-dihydroxyphenyl)-propionic acid derivative can be synthesized by laccase-catalyzed N-coupling of aromatic and aliphatic amines, and (C) the coupling of *p*-hydroquinones with primary aromatic amines by laccases.

Last but not least, laccase finds applications in the synthesis of hormone derivatives (generating dimers or oligomers by the coupling of the reactive radical intermediates). Intra et al. [172] and Nicotra et al. [44] have recently exploited the laccase capabilities to isolate new dimeric derivatives of the hormone β-estradiol (Figure 8A) and of the phytoalexin resveratrol (Figure 8B), respectively. Similarly, laccase oxidation of totarol, and of isoeugenol or coniferyl alcohol gave novel dimeric derivatives [134] and a mixture of dimeric and tetrameric derivatives [173] respectively, whereas an even more complex mixture of products was observed in the oxidation of substituted imidazole (Figure 9A) [126]. These novel substituted imidazoles or oligomerization products (2–4) are applicable for pharmacological purposes. In another study, derivatization of the natural compound 3-(3,4-dihydroxyphenyl)-propionic acid can be achieved by laccase-catalyzed N-coupling of aromatic and aliphatic amines (Figure 9B). The derivatives of this antiviral natural compound 3-(3,4-dihydroxyphenyl)-propionic acid may have interesting pharmaceutical uses. More recently, nuclear amination of *p*-hydroquinones with primary aromatic amines catalyzed by laccases in the presence of O_2_resulted in the formation of the corresponding monoaminated or diaminated quinones [174,175], (Figure 9C).

## Conclusion

The use of laccases in organic synthesis does show as a promising green alternative to the classical chemical oxidation with a wide range of substrates. In the near future, the practical use of fungal laccases for troublesome transformations (digestion of lignocellulose to use as a carbon source; modifications of lignosulfonates for production of emulsifiers, surfactants and adhesives; synthesis of polymers with properties as redox films for bioelectronic devices; synthesis of antibiotics and much more) will expand the need for this biocatalyst. Meanwhile, the development of more robust fungal laccases tailored by protein engineering and the search for environment-friendly mediators along with further research on heterologous expression are significant hurdles that must be overcome.

## Competing interests

The authors declare that they have no competing interests.

## Authors' contributions

AB suggested the topic and got the approval  from the Editor. AK wrote the first draft. MA wrote the second draft, which was revised critically and contributed additional content throughout by AK, AB, FJP, SC and CGB. MA coordinated the final version of the review, which was read and approved by all authors. 
